# Supplementary material for: Antimicrobial Efficacy and Spectrum of Phosphorous-Fluorine Co-Doped TiO2 Nanoparticles on the Foodborne Pathogenic Bacteria Campylobacter jejuni, Salmonella Typhimurium, Enterohaemorrhagic E. coli, Yersinia enterocolitica, Shewanella putrefaciens, Listeria monocytogenes and Staphylococcus aureus
Source: Foods. 2021 Jul 31;10(8):1786. doi: 10.3390/foods10081786 (PMC8391345; doi:10.3390/foods10081786)
Supplement: Supplementary file 1 [file foods-10-01786-s001.zip › foods-1280530-supplementary.pdf]

Supplementary Material

Antimicrobial efficacy and spectrum of Phosphorous-Fluorine co-doped TiO_2 nanoparticles on the foodborne pathogenic bacteria *Campylobacter jejuni*, *Salmonella* Typhimurium, Enterohaemorrhagic *E. coli*, *Yersinia enterocolitica*, *Shewanella putrefaciens, Listeria monocytogenes* and *Staphylococcus aureus*

György Schneider[1], Bettina Schweitzer[1], Anita Steinbach[1], Botond Zsombor Pertics[1], Alysia Cox[2], and László Kőrösi[3]

[1]Department of Medical Microbiology and Immunology, Medical School, University of Pécs, H-7624 Pécs, Szigeti st. 12., Hungary

[2]Department of Biotechnology, Nanophagetherapy Center, Enviroinvest Corporation, Pécs, Kertváros út 2., Hungary

[3]Research Institute for Viticulture and Oenology, University of Pécs, H-7634 Pécs, Pázmány Péter út 4., Hungary

Figure S1: Concentration dependent antibacterial activities of PF-TiO_2 NPs on *Campylobacter jejuni* (a), *Salmonella* Typhimurium (b), Enterohaemorrhagic *E. coli* (c), *Yersinia enterocolitica* (d), *Shewanella putrefaciens* (e), *Listeria monocytogenes* (f) and *Staphylococcus aureus* (g). Experiments were performed under dark conditions, or daylight exposures or UVA irradiations for 30 minutes in 0.5 mg/mL, 0.1 mg/mL, 0.02 mg/mL NP dispersions. The initial living cell numbers were ~10^6 CFU/mL.

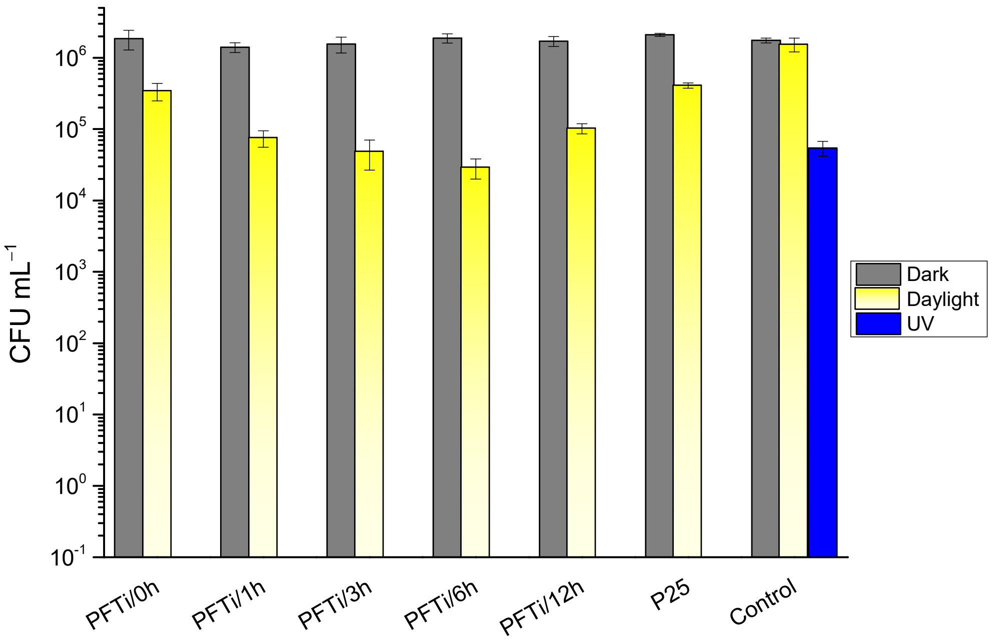

a/1.

C. jejuni; [TiO_2]= 0.5 mg/mL

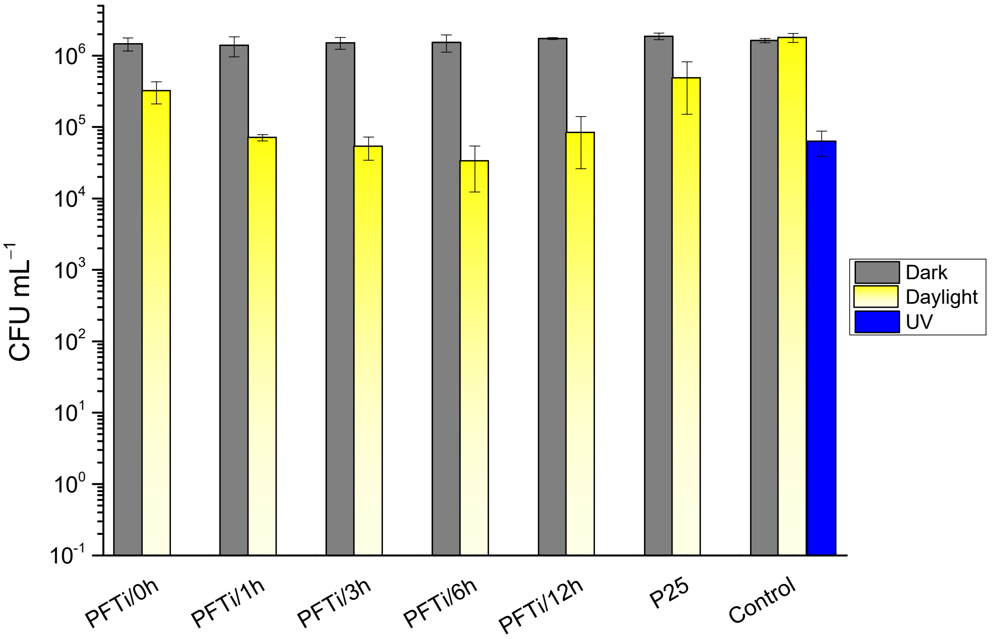

a/2.

C. jejuni; [TiO_2]= 0.1 mg/mL

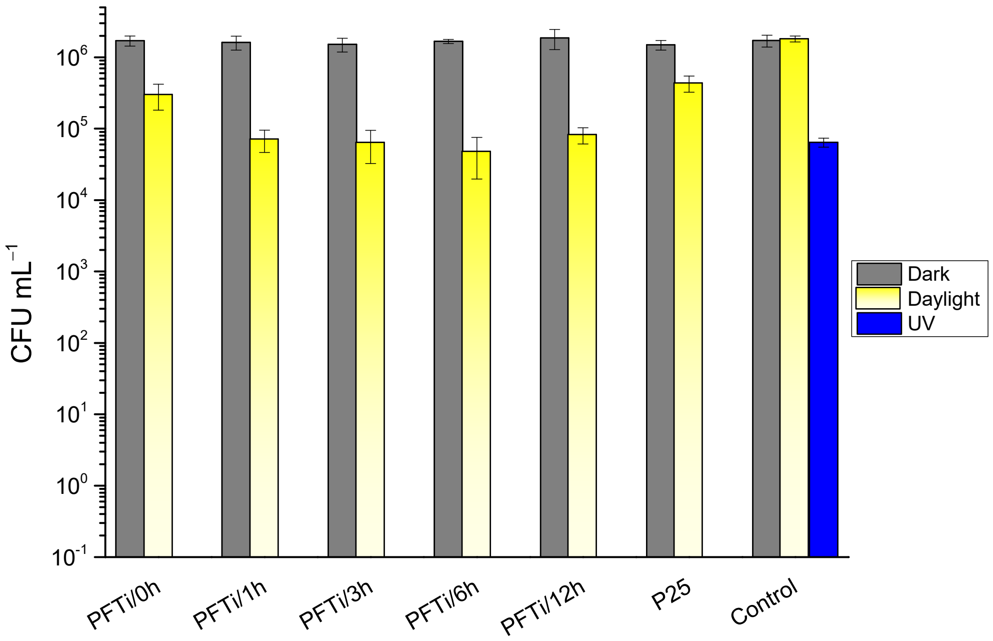

a/3.

C. jejuni; [TiO_2]= 0.02 mg/mL

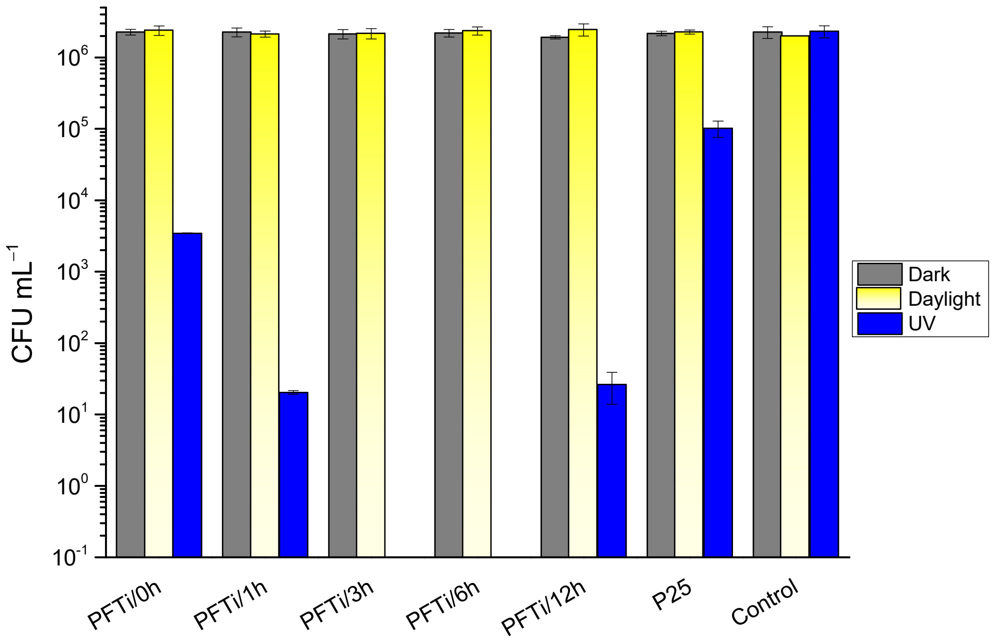

b/1.

Salmonella Typhimurium*;* [TiO_2]= 0.5 mg/mL

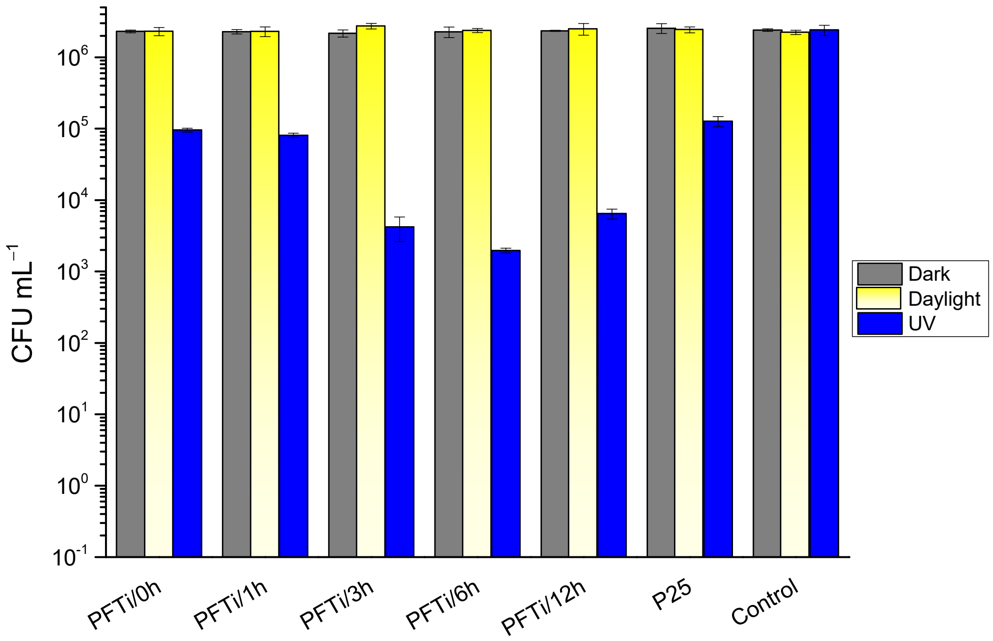

b/2.

Salmonella Typhimurium*;* [TiO_2]= 0.1 mg/mL

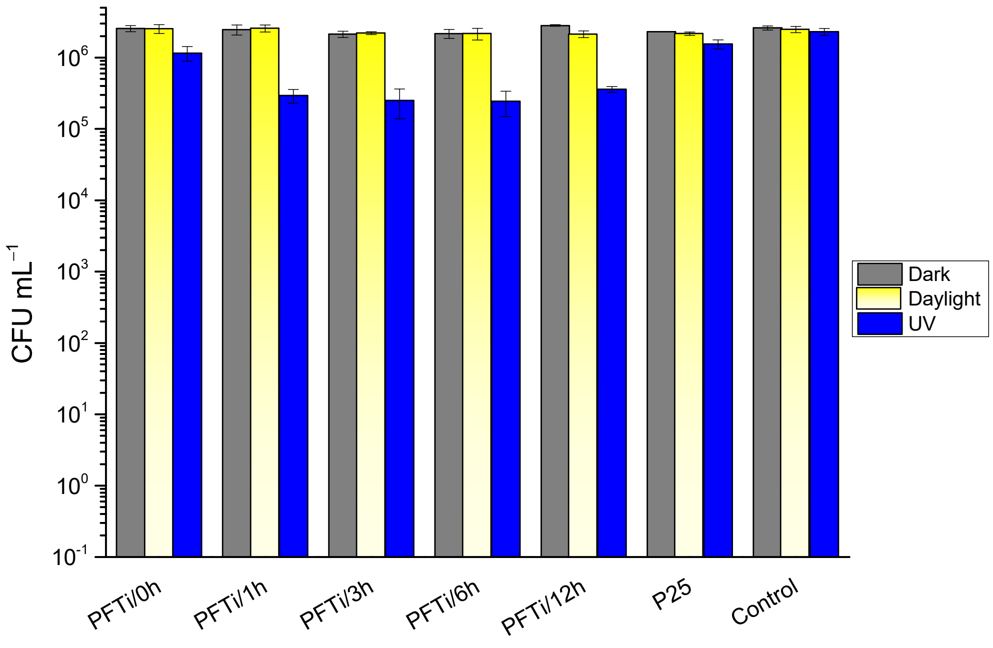

b/3.

Salmonella Typhimurium*;* [TiO_2]= 0.02 mg/mL

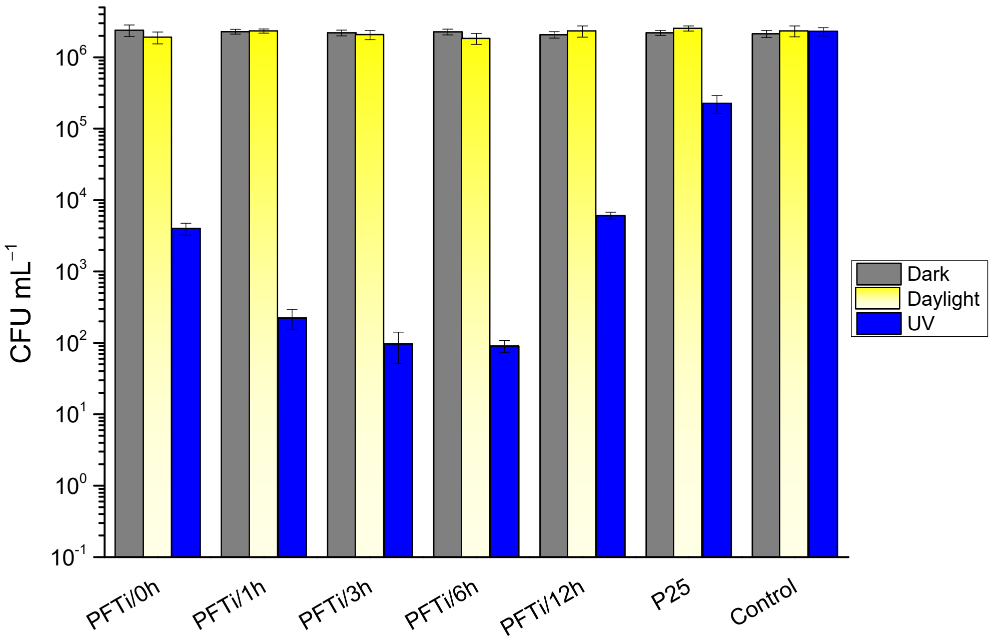

c/1.

EHEC; [TiO_2]= 0.5 mg/mL

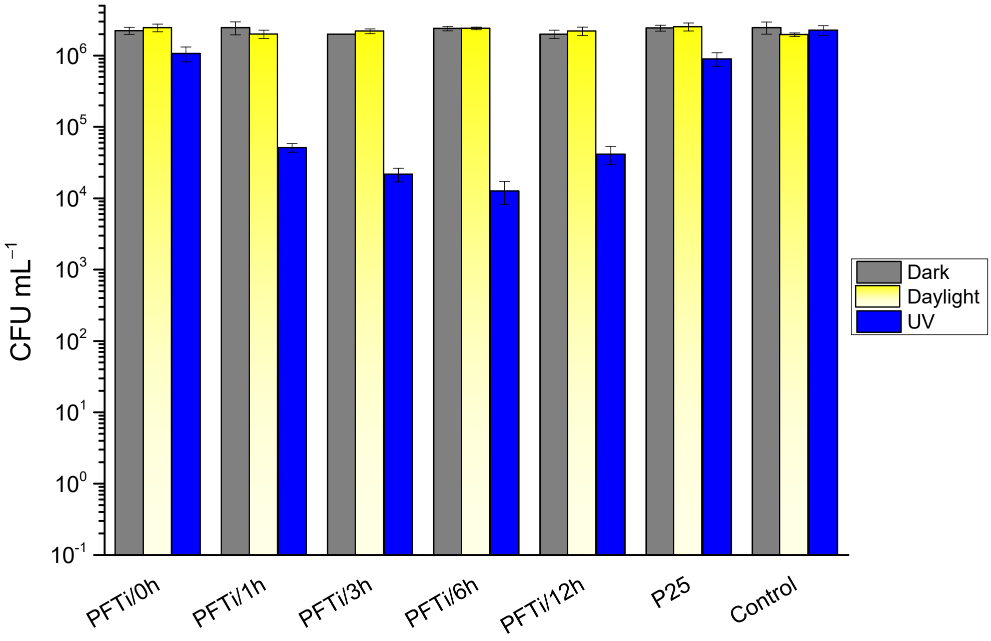

c/2.

EHEC; [TiO_2]= 0.1 mg/mL

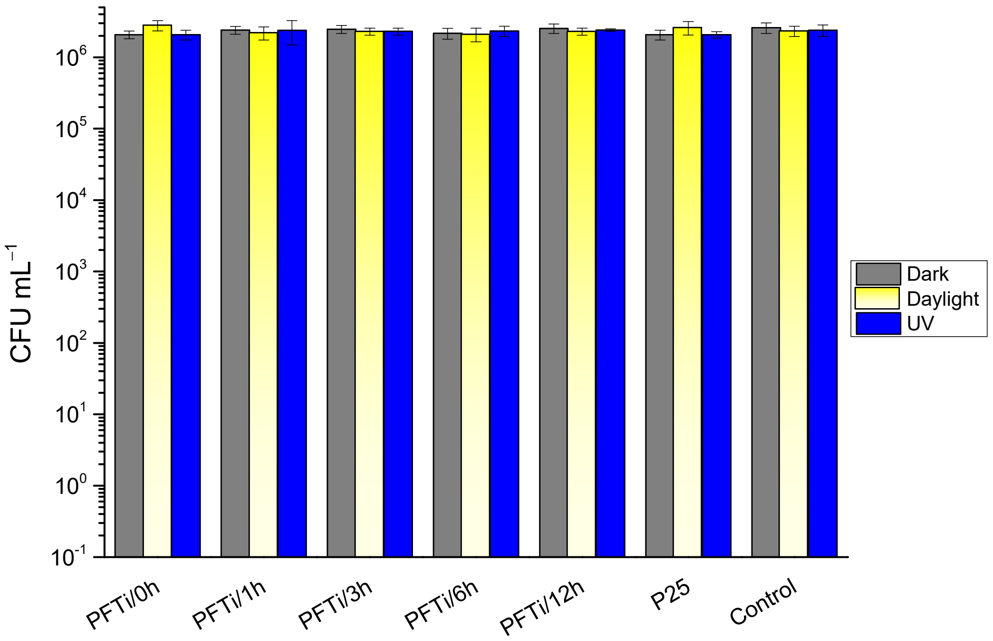

c/3.

EHEC; [TiO_2]= 0.02 mg/mL

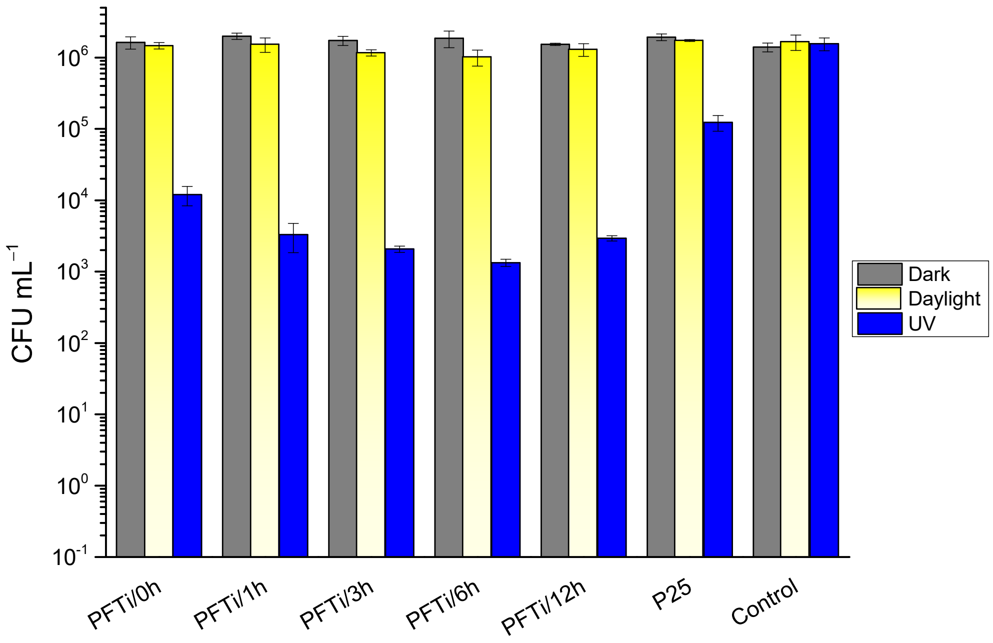

d/1.

Y. eneterocolitica; [TiO_2]= 0.5 mg/mL

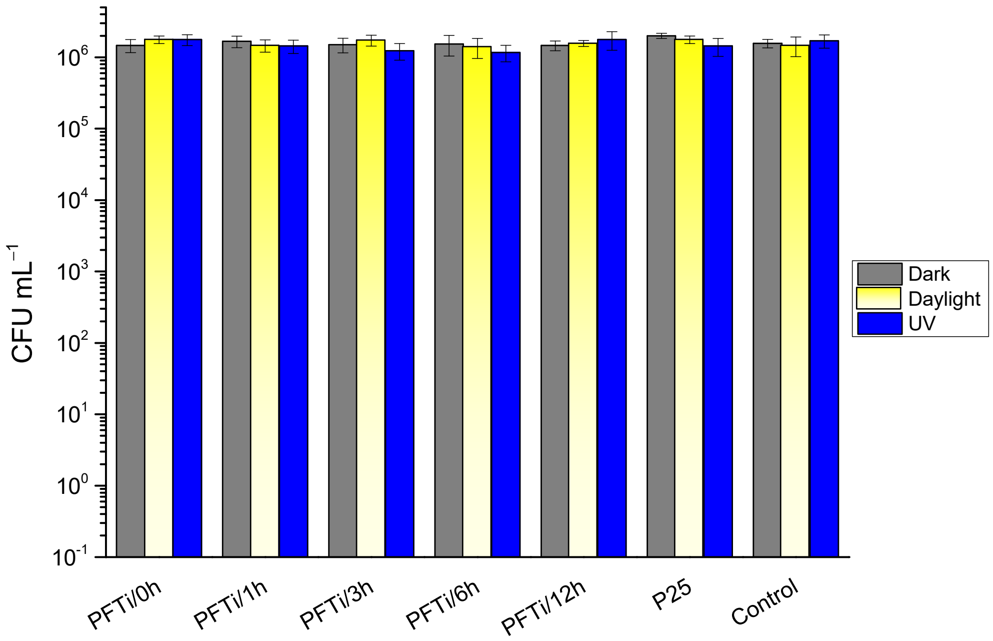

d/2.

Y. eneterocolitica; [TiO_2]= 0.1 mg/mL

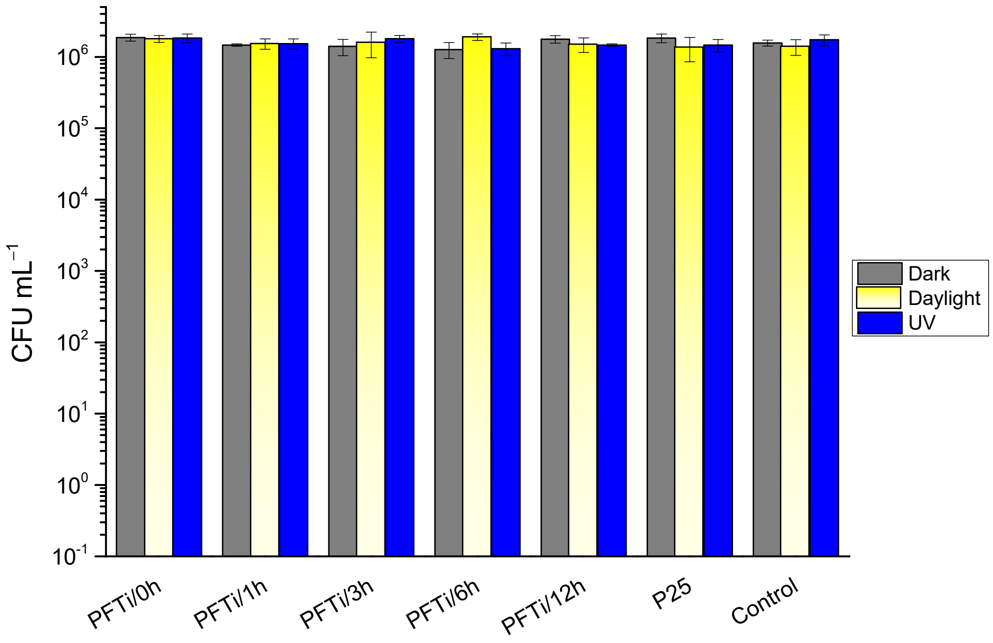

d/3.

Y. eneterocolitica; [TiO_2]= 0.02 mg/mL

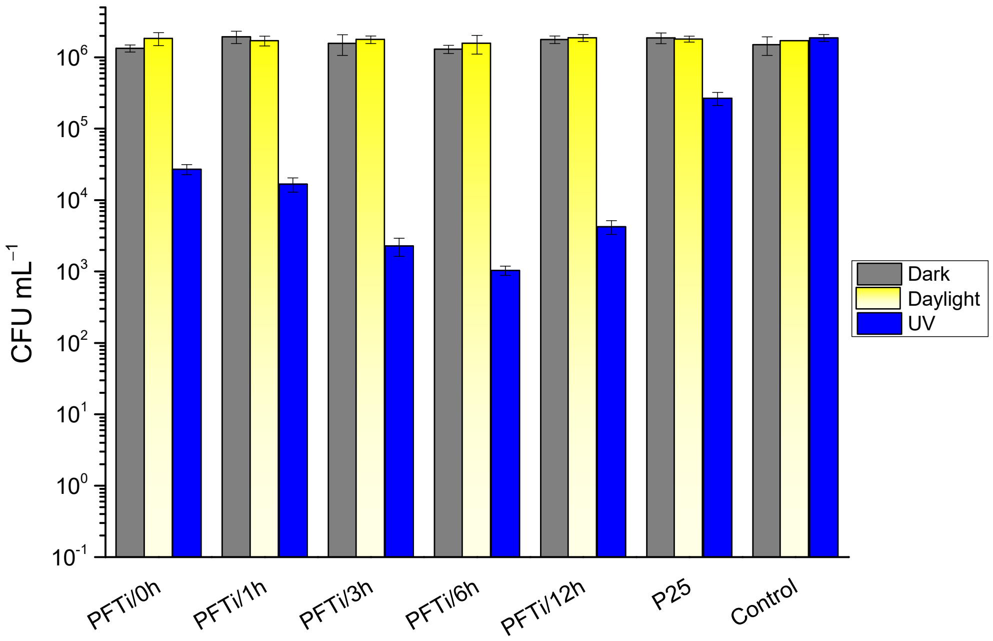

e/1.

S. putrefaciens; [TiO_2]= 0.5 mg/mL

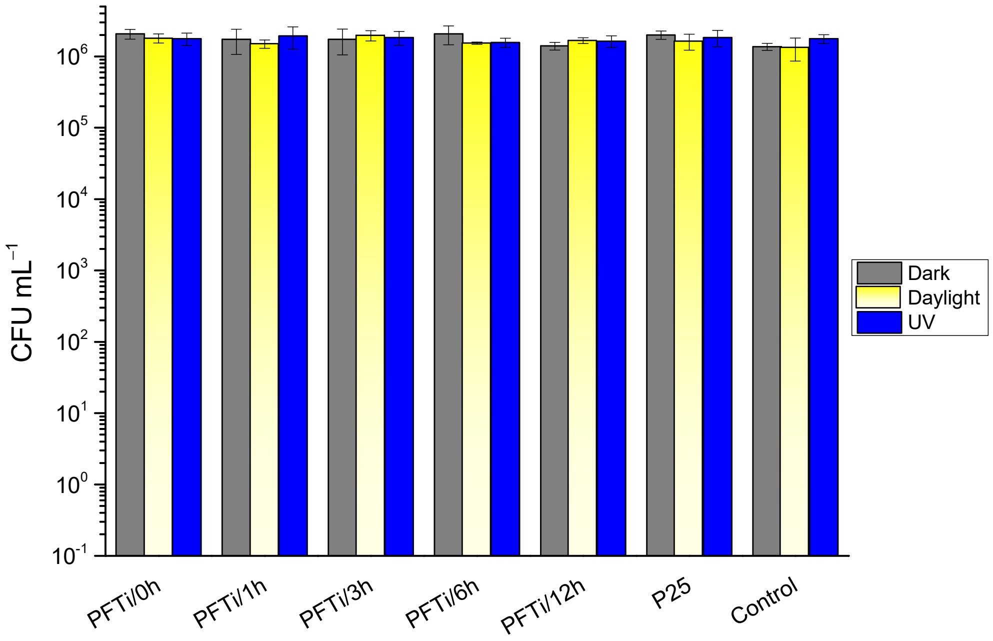

e/2.

S. putrefaciens; [TiO_2]= 0.1 mg/mL

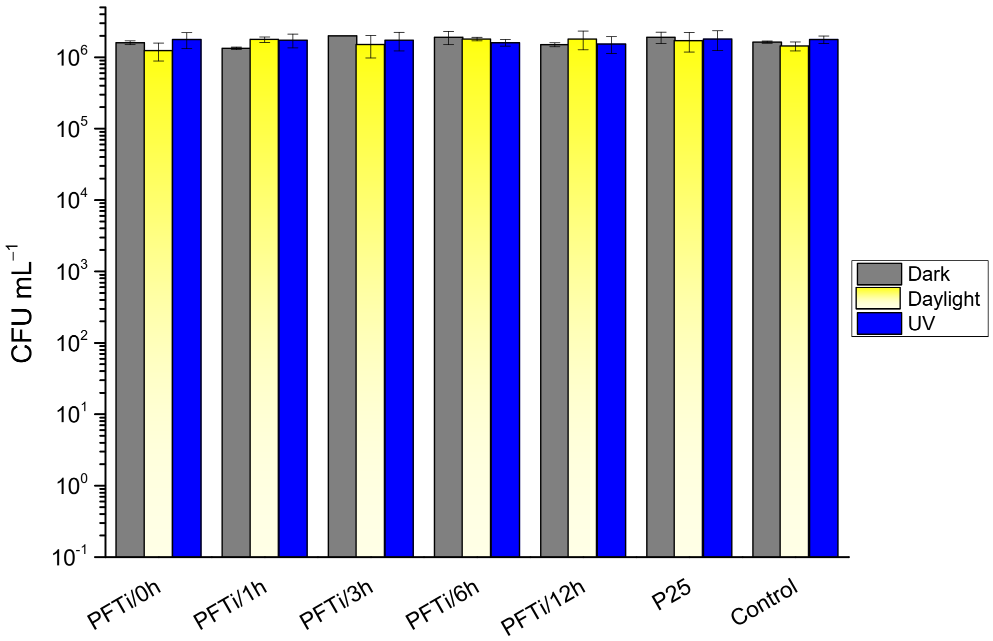

e/3.

S. putrefaciens; [TiO_2]= 0.02 mg/mL

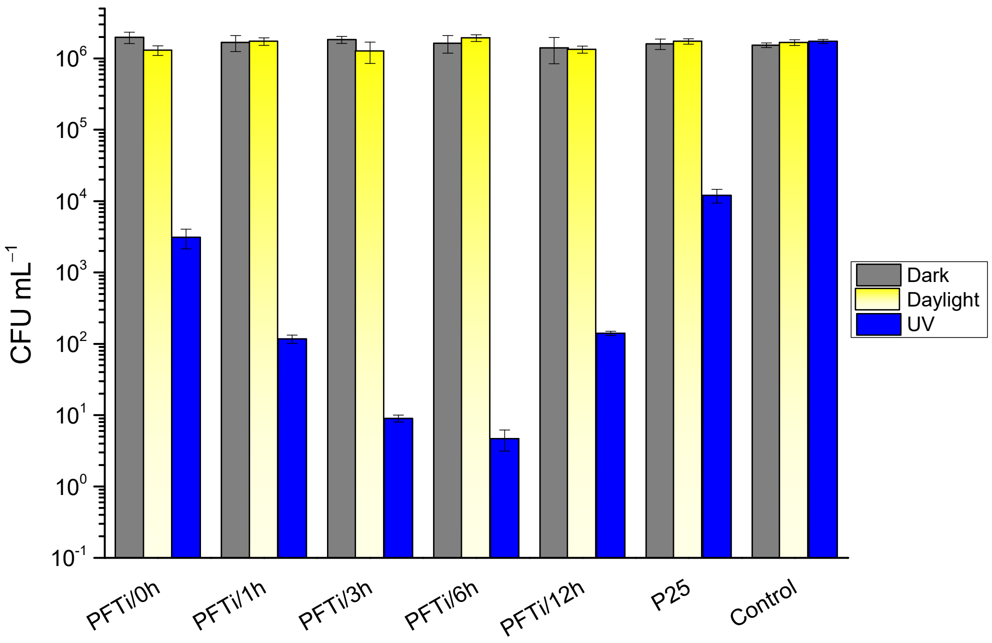

f/1.

L. monocytogenes; [TiO_2]= 0.5 mg/mL

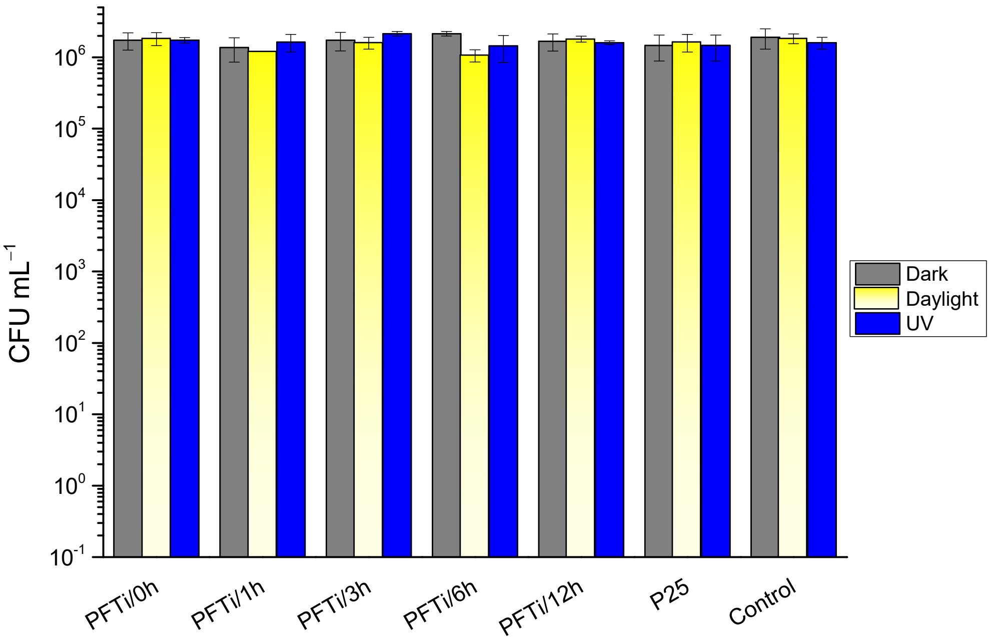

f/2.

L. monocytogenes; [TiO_2]= 0.1 mg/mL

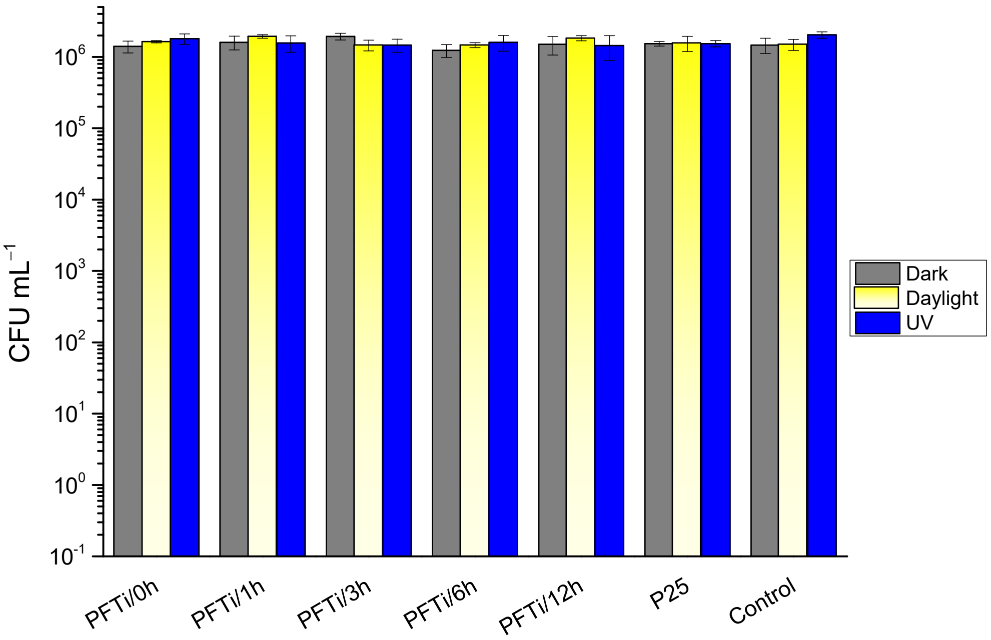

f/3.

L. monocytogenes; [TiO_2]= 0.02 mg/mL

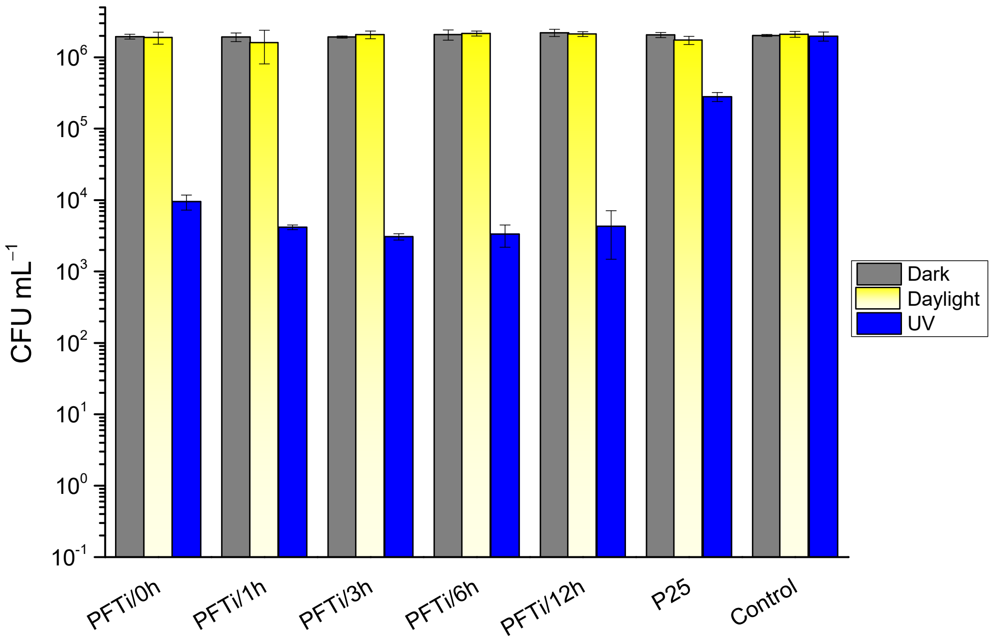

g/1.

S. aureus; [TiO_2]= 0.5 mg/mL

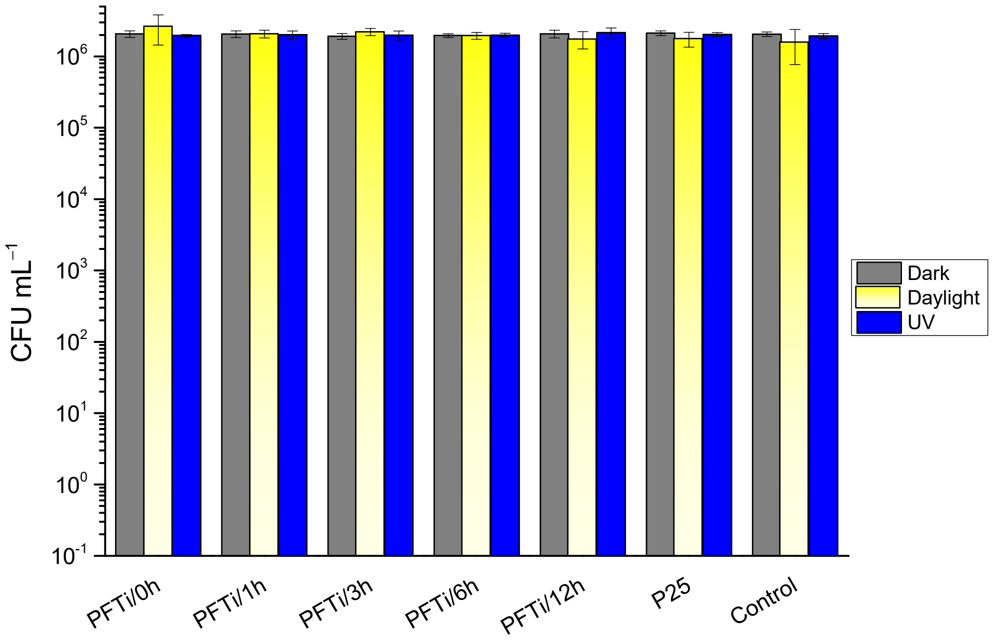

g/2.

S. aureus; [TiO_2]= 0.1 mg/mL

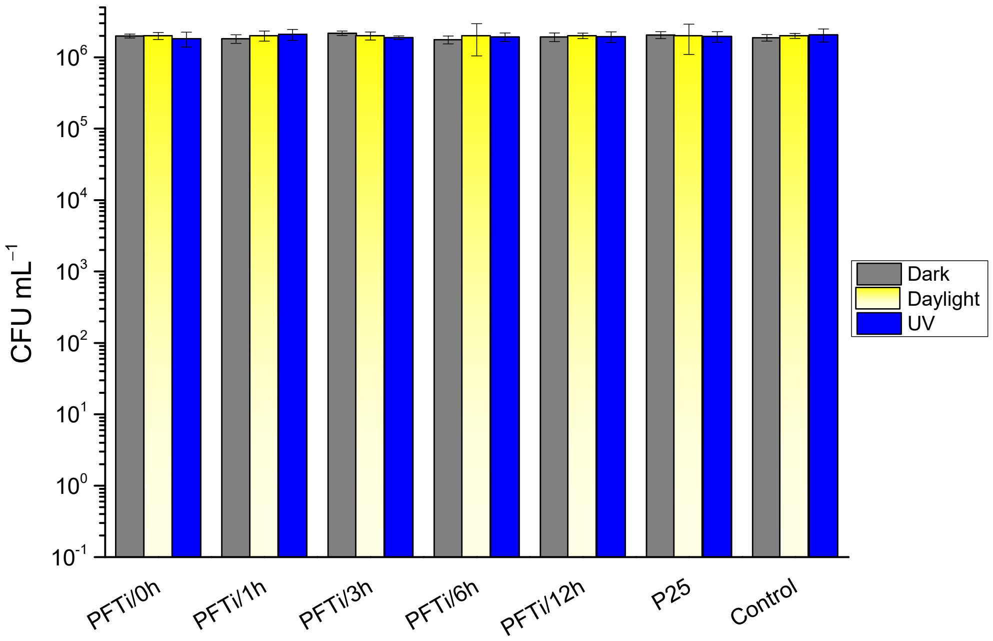

g/3.

S. aureus; [TiO_2]= 0.02 mg/mL
